# On Acquisition Parameters and Processing Techniques for Interparticle Contact Detection in Granular Packings Using Synchrotron Computed Tomography

**DOI:** 10.3390/jimaging8050135

**Published:** 2022-05-12

**Authors:** Fernando Alvarez-Borges, Sharif Ahmed, Robert C. Atwood

**Affiliations:** 1 Diamond Light Source Ltd., Harwell Science & Innovation Campus, Didcot OX11 0DE, UK; sharif.ahmed@diamond.ac.uk (S.A.); robert.atwood@diamond.ac.uk (R.C.A.); 2 Faculty of Engineering and Physical Sciences, University of Southampton, Southampton SO17 1BJ, UK

**Keywords:** contact detection, computed tomography, segmentation, soil, granular materials

## Abstract

X-ray computed tomography (XCT) is regularly employed in geomechanics to non-destructively measure the solid and pore fractions of soil and rock from reconstructed 3D images. With the increasing availability of high-resolution XCT imaging systems, researchers now seek to measure microfabric parameters such as the number and area of interparticle contacts, which can then be used to inform soil behaviour modelling techniques. However, recent research has evidenced that conventional image processing methods consistently overestimate the number and area of interparticle contacts, mainly due to acquisition-driven image artefacts. The present study seeks to address this issue by systematically assessing the role of XCT acquisition parameters in the accurate detection of interparticle contacts. To this end, synchrotron XCT has been applied to a hexagonal close-packed arrangement of glass pellets with and without a prescribed separation between lattice layers. Different values for the number of projections, exposure time, and rotation range have been evaluated. Conventional global grey value thresholding and novel U-Net segmentation methods have been assessed, followed by local refinements at the presumptive contacts, as per recently proposed contact detection routines. The effect of the different acquisition set-ups and segmentation techniques on contact detection performance is presented and discussed, and optimised workflows are proposed.

## 1. Introduction

Since its introduction to the field of geomechanics [1], X-ray computed tomography (XCT) has been employed to quantify the solid and void fractions present in soil and rock samples. Bulk parameters such as porosity and void ratio are routinely derived from these measurements (see e.g., [1,2,3,4]). With the improvement of XCT systems and the associated increase in spatial resolution of the reconstructed 3D images, researchers now also seek to measure fabric-level parameters, such as particle and/or pore size, shape, and organisation [5,6,7]. In particular, the number, area and orientation of interparticle contacts are coveted fabric quantities that may be retrieved from XCT data. These parameters can then be used to, for example, analyse the micromechanical behaviour of geomaterials [8,9,10] and inform or assess computer modelling methods [11,12,13].

The retrieval of interparticle contact information from reconstructed XCT images involves the classification of voxels into the individual soil grains contained in the image, in other words, image segmentation. A typical segmentation workflow comprises the binarisation of the image into solid and pore labels followed by the subdivision of the solid label into individual soil grains. The former is commonly carried out by thresholding while the latter is often performed using a watershed method (see, e.g., [13,14,15]). Thresholding involves the selection and labelling of voxels exhibiting grey level values above or below a given number. The watershed method interprets images as topographical maps where voxel values correspond to different ‘elevations’ that define ‘catchment basins’. Basins for individual soil grains are often identified, or ‘marked’, using the local minima resulting from applying a distance transform to the binarised image. These markers are then morphologically dilated until contact with neighbouring markers is established, thus ‘filling-up’ the basin. The catchment basins image is then overlaid with the original binarised input to remove background voxels and obtain the segmentation output, where every soil grain is identified by a different label. Finally, interparticle contacts are measured by finding the voxels at the boundary between neighbouring grain labels.

**Figure 1:**
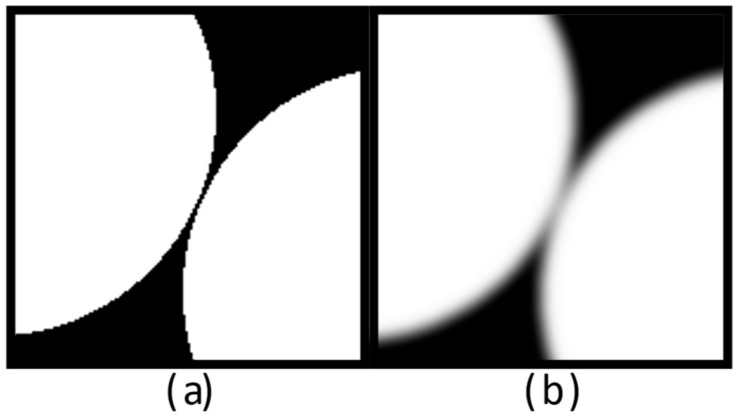
Representation of two non-contacting particles (**a**) and the effect of simulated partial volume blur (**b**), resulting in an increase in greyscale tone of the pixels in the gap between particles.

While this procedure is common practice and of relatively straightforward implementation, recent research has evidenced that it consistently overestimates the number and area of interparticle contacts [16,17]. This appears to be predominantly driven by the partial volume effect, which results from the grey value representation of features smaller than the XCT system voxel size as the average of their X-ray attenuation [18,19]. The grain surface curvature (or roundness/angularity) and texture (or roughness) are features that are often smaller than the voxel size, and thus, the precise location of grain boundaries is frequently lost due to the blurriness introduced by the partial volume effect [18]. The addition of the partial volume blur of two grain surfaces in close proximity then leads to spurious contacts in the reconstructed XCT image [16,17] (see also [20,21]). This is presented schematically in Figure 1.

These previous investigations have delivered valuable insight on contact detection issues. However, limited attention has been given to the role of XCT acquisition parameters in the introduction of noise and image artefacts that may hamper interparticle contact recognition. For instance, Wiebicke et al. [17] used synthetic and very high-resolution data with a limited field-of-view (FOV) to which image binning (combining the output of two or more pixels into one) and Gaussian noise treatments were applied afterwards to simulate ‘realistic’ acquisition conditions. While this approach is sensible for consistency and replicability purposes, the added noise may differ from truly realistic conditions and disregard other acquisition artefacts (zingers, rings, beam flux effects, etc.; see, e.g., [18,21,22,23,24]), especially considering the enormous variability in XCT systems and setups. Kiekens et al. [20] used different X-ray energies to scan a non-granular object to investigate edge detection, but the role of the number of projections and the exposure time per projection was beyond the scope of their work. The work of Villarraga-Gómez and Smith [25] showed that significant reduction in the number of projections does not lead to large errors in linear measurements of workpieces, but that even a minor projection undersampling leads to large area and volume measurement errors. The effect of these errors in contact detection was not considered, as their investigation focused on industrial component manufacturing rather than granular materials. Thus, a systematic assessment of the role of the number of projections, exposure time, and angular range for large FOV XCT measurement of interparticle contacts in granular packings remains absent in the literature, to the authors’ knowledge.

The influence of the segmentation technique on interparticle contact detection has also received limited attention, as most previous works have based their studies on thresholding approaches. Recent investigations have shown that segmentation methods based on convolutional neural networks (CNNs) may deliver a higher accuracy than conventional thresholding techniques [23,24]. CNNs are a class of deep neural networks consisting of a series of levels or ‘layers’ where filters or ‘kernels’ are applied (‘convolved’) to extract image features, progressively optimizing (‘learning’) filter parameters and order of application [26,27]. However, the impact of these seemingly improved segmentation methods on interparticle contact detection has not yet been thoroughly investigated.

The present paper aims to deliver insight into these issues by methodically assessing the role of basic synchrotron XCT acquisition parameters, namely, the number of projections, exposure time, angular range, and FOV extension by sample offsetting, in the accurate detection of the number and area of interparticle contacts of a granular sample. The novel contact detection technique proposed by Wiebicke et al. [17] is used for this purpose, but the effect of segmentation accuracy is investigated by using both a CNN-based approach and conventional thresholding, as in the original method. An improved workflow for contact detection is then proposed from the analysis of results.

## 2. Materials and Methods

### 2.1. Sample Description

**Figure 2:**
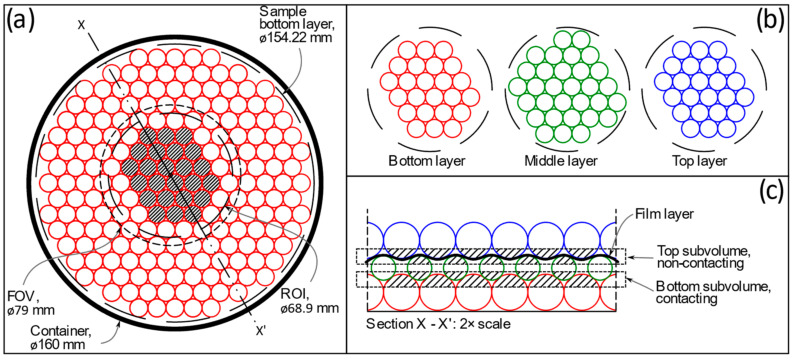
Pellet sample schematic: (**a**) plane view of bottom layer with pellets used in the analysis indicated by hatched region; (**b**) plane view of bottom, middle and top layers of pellets within the central ROI used in the analysis; (**c**) X-X’ vertical cross section through central ROI showing the top and bottom subvolumes, the film layer separating the middle and top lattices, and pellets used in the analysis indicated by hatched regions.

The granular sample used in the present study was composed of soda-lime glass pellets with a nominal diameter of 10 mm. This particle size and shape was chosen to facilitate sample preparation and promote partial volume blurring, and is not representative of typical soil. The pellets were placed in three layers (or lattices) in a hexagonal close-packed (HCP) arrangement inside a flat-ended cylindrical polymethyl methacrylate container of 160 mm in internal diameter, as depicted in Figure 2a. A closed-cell polyethylene foam mould was used to provide lateral support to the bottom layer of pellets inside the container and maintain the hexagonal shape of the lattice. This layer contained a total of 458 pellets and could be circumscribed by a circle of approximately 154.22 mm in diameter. The middle and top layers were progressively smaller, thus forming a truncated pyramid. These layers contained 377 and 302 pellets, respectively. Each pellet layer was horizontally circumscribable by circles with diameters of 146.14 mm and 134.9 mm, respectively.

A separation between the middle and top lattice was created by means of a triple layer of polyethylene (PE) film (commercial food wrap or ‘cling film’) with a total thickness of about 38 μm, as schematically shown in Figure 2c. Thus, no interparticle (grain to grain) contacts occurred between middle and top layer pellets. The differences in density (*ρ*) and X-ray attenuation (μ) of polyethylene and soda-lime glass (*ρ* ≈ 0.92 g/cm^3^ vs. *ρ* ≈ 2.50 g/cm^3^, respectively [28,29]; and μ ≈ 0.14 cm^−1^ vs. μ ≈ 0.66 cm^−1^ at 145 keV, respectively [30,31]) meant that both materials could be easily differentiated in the reconstructed XCT images. No load besides the self-weight of the overlaying pellet layer was applied to the PE film to minimise volumetric deformations that could compromise its detectability via XCT. In this way, the sample contained readily identifiable examples of contacting and non-contacting particles from which to assess the accuracy of XCT-based contact detection routines.

### 2.2. Synchrotron X-ray Computed Tomography

XCT comprises the acquisition of 2D X-ray radiographies (projections) from prescribed angular positions within an angular range, often 180° (half rotation) or 360° (full rotation), followed by the mathematical reconstruction of a 3D image of the scanned object using said projections. In synchrotron XCT, the X-ray beam is generated by the acceleration of electrons to relativistic speeds, and exhibits high intensity, high coherence, and low divergence [32]. Additionally, the energy spectrum can usually be tuned to achieve monochromatic conditions. Further details on the fundamentals of XCT imaging may be found in, e.g., [22,33].

For the present study, synchrotron XCT was performed using Experimental Hutch 2 of beamline I12-JEEP at Diamond Light Source. A monochromatic X-ray beam at 145 keV was used. A large FOV detector system consisting of two scintillator-coupled pco.EDGE 5.5 sCMOS cameras each fitted with an optics module was employed. This system is described in [34] (see also [35]). The detector delivered a pixel length of 21 μm and a FOV of 79 mm in width by 25 mm in height, approximately. During scanning, X-ray projections were acquired while the sample rotated on the tomography stage. A total of eight scans were carried out. For each scan, the number of projections, exposure time, and rotation range were varied, as presented in Table 1. Series A encompassed scans acquired using 0.4 s exposure time per projection. Scans A1–A3 were conventional 180° rotation synchrotron acquisition scans, while scans A4, A5 were full 360° rotation acquisitions. Scan A4 aimed to assess the effect of recording projections through a full rotation, as commonly done in laboratory-based XCT, versus the conventional 180° acquisition used in synchrotron XCT (i.e., scan A3). Scan A5 involved a technique to increase the imaging FOV, which is discussed later. Series B encompassed scans with acquisition times per projection varying from 0.2 to 0.05 s. Scan B1 aimed to assess whether acquiring projections throughout a full sample rotation but halving the exposure time per projection resulted in any advantages compared with conventional half-rotation scan A2 with twice the exposure time per projection and the same total X-ray exposure time. Scans B2 and B3 are conventional half-rotation scans where the exposure time per projections was halved with regards to the previous scan.

Sample dimensions exceed the horizontal size of the FOV achievable by the detector system in a standard scan set-up, as depicted in Figure 2a. Thus, most scans were ‘local’, that is, they covered a central region rather than the entire sample. This approach was used to image the pellets at the centre of the sample (i.e., non-boundary pellets) which exhibit all nominal interparticle contacts for an HCP lattice, minus those suppressed by the PE film. An exception is the non-standard scan A5, for which the FOV was extended by offsetting the sample from the centre of rotation (COR) and acquiring projections throughout a 360° range—thus capturing the entire sample. This is known as the ‘half-acquisition’ or ‘offset scanning’ technique, where half of the sample width is radiographed per 180° rotation [33,36,37]. This was done to assess the effect of this acquisition technique on contact detection, if any, and is the reason behind the preparation of a sample considerably wider than the FOV of the system under conventional scanning conditions.

The characteristics of the beam profile resulted in higher X-ray flux being available at the centre of the beam, with flux reducing towards the edges. This, in addition to truncation artefacts caused by beam attenuation produced by sample regions out of the FOV [38], led to lower reconstructed image quality at the periphery of the FOV. Thus, the pellets at the periphery of the FOV were omitted from the analysis, retaining only those at the centre of the image, depicted in Figure 2b. The region of interest (ROI) containing these ‘central’ pellets had a diameter of 68.9 mm (3280 voxels), approximately. For consistency, the same ROI pellets were extracted and analysed in the half-acquisition scan A5 (Table 1), which had a much larger FOV (162.9 mm wide). The central ROI contained 79 pellets, with 24 pellets in the bottom and top layer each.

Between 721 and 3601 projections per 180° rotation were obtained for each scan using a fly-scan technique where the sample rotates continuously during projection acquisition. The number of projections represented between 14 and 70% of the Nyquist optimum of 5152 projections for the 68.9 mm ROI at the centre of the FOV (0.5 × π × 3280 ROI voxel width ≈ 5152 projections; [33]). Based on Villarraga-Gómez and Smith [25], it was surmised that increasing the number of projections beyond 70% of the Nyquist optimum would have delivered limited improvements in reconstructed image quality. It would have also significantly increased acquisition times, thus limiting the number of scans that could be performed. Projection undersampling at 60 to 70% of the Nyquist optimum is often found in in the literature [39]. The exposure time per projection ranged between 0.05 s and 0.4 s (Table 1).

Tomographic reconstruction was carried out using Savu [40]. The processing pipeline included automated zinger (speckle) removal [41], ring artefact suppression [42], and COR determination [43], followed by reconstruction using filtered back-projection (FBP) [44,45]. For full-rotation and half-acquisition scans, FBP was performed by converting the 360° sinograms into 180° via stitching of the top half with the bottom half. The overlap strip was merged using a ‘ramp’ (or ‘tapper’) linear weighting method. Further details on FBP reconstruction are available in, e.g., [22,33].

After reconstruction, the ROI was subdivided into ‘bottom’ and ‘top’ subvolumes to reduce computing times, as shown in Figure 2c. As presented in this figure, the bottom subvolume overlapped the bottom and middle lattice to enclose the interparticle contacts amongst them. Likewise, the top subvolume intersected the middle and top layers to encompass the PE film-separated non-contacting pellet boundaries between both layers.

### 2.3. Image Segmentation

Two binarisation techniques were applied to the reconstructed XCT data: thresholding, described previously in Section 1, and CNN-based segmentation using U-Nets [46]. Prior to either binarisation routine, the reconstructed subvolumes were downscaled from 32 bit to 8 bit to expedite computing times, followed by the application of median (kernel = 2) and anisotropic diffusion (diffusion threshold = 100; iterations = 2) filters using Avizo Lite^®^ (Thermo Fisher Scientific, Waltham, MA, USA). to reduce image noise and improve segmentation outcomes. The filtered bottom and top subvolumes for each scan are available in [47].

U-Nets are a class of CNN composed of a downsampling and upsampling paths. As with other CNNs, filters are applied during downsampling or ‘contraction’, reducing spatial resolution while retrieving features. During upsampling or ‘expansion’, spatial and feature information are recombined using direct connections from each level of the downsampling pathway, returning the label image.

U-Net binarisation was carried out using RootPainter [48,49], which has been shown to deliver highly accurate segmentations of XCT data, including images of granular materials, and is straightforward to implement [50,51]. RootPainter is a client-server application for binary segmentation in which the user labels 2D images by manually annotating (‘painting’) foreground (feature of interest) and background (everything else) regions using the client’s graphical user interface. The server then uses the label images for training, with one out of five images used for validation. The server returns a segmentation model after each training epoch (that is, after passing all available training images through the U-Net), and saves it if its accuracy metric (balanced F-score, or F_1_; see also [52]) is greater than that of any previously saved model. Once a U-Net model is available, the user engages in interactive corrections of the segmentation output (overlaid over the original input image) by painting over erroneously segmented pixels with the correct foreground or background label. These label images are added to the training and validation datasets and used in subsequent training epochs.

U-Net binarisation was performed on the horizontal slices of the top and bottom subvolumes. A total of 50 slices was annotated for training, with the glass pellets labelled as foreground. The slices were pseudo-randomly selected from both the bottom and top subvolume. After annotation, the software was allowed to train until reaching the maximum number of 60 epochs without accuracy improvement. The data received no additional treatment (e.g., morphological operations) after U-Net binarisation.

**Figure 3:**
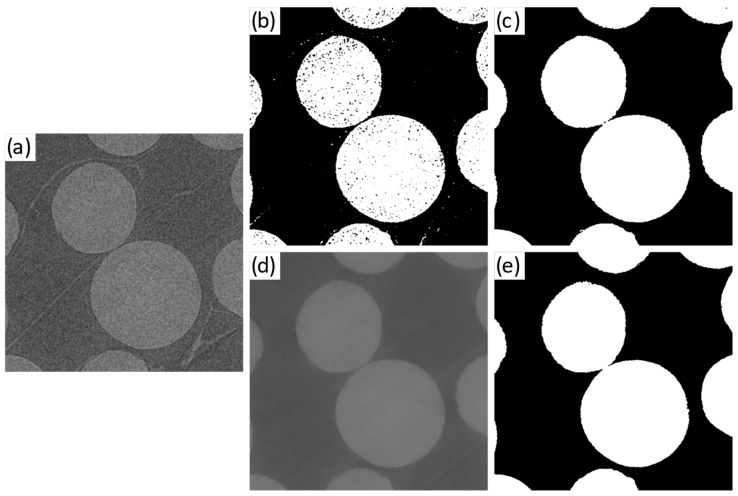
Section of horizontal XCT slice for scan B2 depicting two PE film-separated pellets: (**a**) after median and anisotropic diffusion filtering; (**b**) binarised using conventional global thresholding; (**c**) after morphological corrections; (**d**) after non-local means filtering; (**e**) binarised by applying conventional global thresholding to the non-local means filtered image.

Conventional global thresholding binarisation was carried out using the Interactive Thresholding tool in Avizo Lite^®^. The threshold value was selected by assessing progressively higher threshold values until one that retained most of the pellet volume and minimised non-pellet voxels was found. Such approach delivered higher accuracy results than alternatives such as the Otsu method [53], due to the histogram skewing effect induced by the peripheral regions affected by the beam flux profile and truncation effects. However, conventional thresholding could yield a ‘noisy’ binary image with ‘holes’ (background voxel islands within the foreground label) and ‘spots’ (foreground voxel islands within the background label) that varied in size and number with the acquisition set-up used, as exemplified in Figure 3a,b. Such binary images could not be adequately separated into individual pellets for contact analysis, as additional labels would be created for some of the noise spots. Two alternatives were considered to mitigate this: the application of a non-local means filter [54] prior to thresholding, or the use of morphological closing and opening after thresholding binarisation (both of them implemented using Avizo Lite^®^ Both alternatives were anticipated to affect interparticle contact detection; the forme{r by blurring particle edges (and thus compounding the partial volume blur; see Figure 1), and the latter by introducing slight changes to the geometry of the particle. Figure 3c–e compares the use of both methods. The non-local means filter used spatial and intensity standard deviations of 5 and 0.2, respectively, and a search window and local neighbourhood size of 10 and 3 pixels, in each case. Morphological closing and opening were preformed using a cubical 3D structural element of one voxel in length. Morphological corrections were preceded by the application of a ‘fill holes’ procedure where background voxel islands within the foreground label were suppressed. As shown in Figure 3, both approaches deliver comparable results, with the non-local means filter method qualitatively exhibiting a marginally larger spurious contact area. For computational efficiency, morphological corrections were applied in place of non-local means filtering. While this step could have been omitted for some of the higher-quality datasets, the procedure was applied to all scans for consistency. The implications of the use of morphological corrections are discussed in Section 4.

Pellets were individually segmented (that is, labelled) by applying the Avizo Lite^®^ Separate Objects tool to the binarised output from each technique. This tool essentially applied a 3D watershed routine to the binary images (using chamfer approximations, 26-connectivity, and H-maxima of 48). This did not entail any changes in to the binarised data (for instance, the number and area of interparticle contacts) other than the assignment of a label number to each pellet. Similar labelling methods have been used by, e.g., Alshibli et al. [6] and Druckrey et al. [15].

### 2.4. Interparticle Contact Measurements and Local Refinement Technique

The contact detection method recently proposed by Wiebicke et al. [17], here referred to as ‘local refinement technique’, was deployed in the present study. In its original presentation, this technique consists in:

1. The binarisation of the target reconstructed XCT volume using a thresholding approach, i.e., the application of a single global threshold value to separate grains from pores in the 3D image (see Section 2.3).
2. The labelling of the binarised image to separate each individual grain.
3. The identification of presumptive interparticle contacts using the labelled volume by finding neighbouring voxels of different grain labels and extraction of interparticle contact areas (i.e., the contacting voxels).
4. The retrieval of contacting voxels on the original greyscale volume, followed by the binary segmentation of these grey value voxels using a higher threshold value than the original global threshold, termed ‘local threshold’.
5. Reassessment of contacts after local thresholding. The rest of the labelled image (obtained in step 2) receives no further treatment.

This technique assumes that the global thresholding method commonly used to binarize the solid fraction of an XCT volume of a granular material will very often select voxels whose grey values result from the addition of the partial volume blur of grain boundaries in close proximity to each other, but that are otherwise not in contact (Section 1; see Figure 1). This occurs because the addition of the partial volume blur results in grey tones that are closer to those of the solid fraction than those of pore space. The higher local threshold applied to the presumptive contacts is aimed at overcoming this by excluding spurious contacts with lower grey values than the actual solid fraction.

Wiebicke et al. [17] recognised that the selection of an appropriate local threshold value is difficult. If the local threshold is too high, true interparticle contacts may be removed; if said value is too low, spurious contacts may remain. In their work, they found that a local threshold around 30% higher than the global threshold delivered suitable results for scans of high-precision spheres. For a spheroidal calcareous sand, a local threshold around 20% higher than the global threshold seemed adequate [55]. For an angular sand, the presence of sharp edges reduced the partial volume effect and increased the risk of suppressing true contacts, and thus, an appropriate local threshold value was not proposed.

As much uncertainty remains on the accurate selection of the local threshold parameter, values between 1 and 1.2 times the global threshold value used for thresholding binarisation were assessed in the present study (in increments of 0.05). These coefficients are hereafter referred to as ‘local threshold factors’, or LTFs. The local refinement technique was applied to the labelled data produced by both global thresholding and U-Net segmentation methods (see Section 2.3). When applied to the former, presumptive contacts were first extracted using the morphologically post-processed label image, followed by the retrieval of contact regions on the greyscale volume and the application of the target local threshold value, including LTF = 1.0. In this way, the contacts obtained after the use of LTF = 1.0 were considered to be the same as those present after global thresholding but before the application of morphological operations. When applied to the U-Net segmented data, the same local thresholds derived from applying LTFs to the optimal global threshold values were applied to presumptive contacts extracted from the labelled data, on which no morphological operations were carried out. Thus, the number and location of contacts on which the local assessment technique was applied depended on the number and location of apparent contacts present in the labelled image.

All contact detection procedures were implemented using the SPAM python library [56], which includes dedicated routines for this purpose (see [57]).

## 3. Results

**Figure 4:**
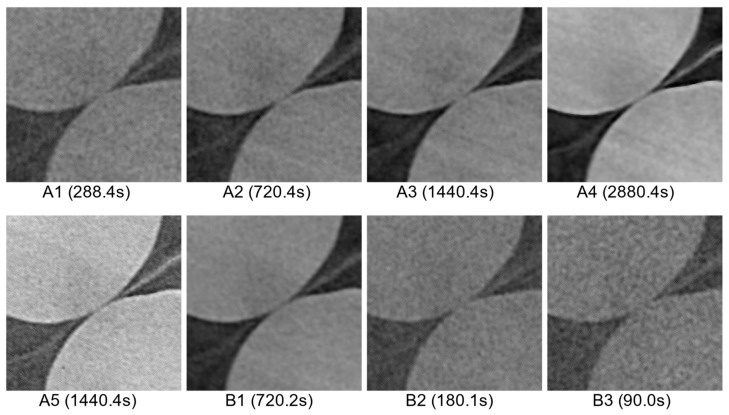
Section of horizontal XCT slices depicting two non-contacting glass pellets separated by a layer of PE film (seen as a diagonal streak) for different acquisition set-ups (Table 1). Total X-ray exposure time for each scan is shown in parenthesis.

Figure 4 presents a section of a central slice extracted from each reconstructed XCT dataset depicting two pellets in close proximity to each other, but not in contact. These pellets correspond to the film-separated top subvolume. The effect of the use of different acquisition parameters can be clearly noted. Scans with larger overall X-ray exposure times deliver clearly distinguishable interparticle boundaries (e.g., scans A4, A5). In contrast, particle separation is less discernible for scans acquired with lower exposure periods, despite all scans having the same spatial resolution. Thus, this figure qualitatively demonstrates how the different acquisition parameters resulted in different greyscale tones for the film-separated interparticle boundary, as well as for the grain and pore materials.

### 3.1. Effect of Number of Projections on Contact Measurements

**Figure 5:**
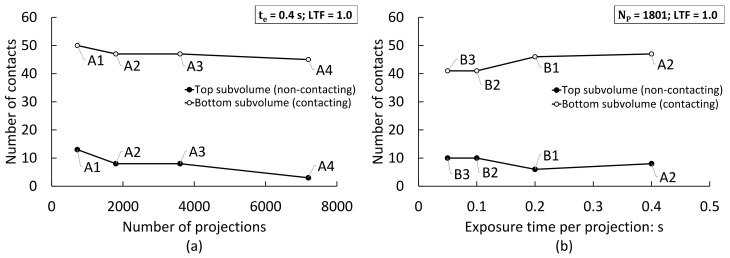
Number of contacts found in XCT data with (top subvolume) and without (bottom subvolume) a prescribed interparticle separation: (**a**) with different number of projections and using a constant exposure time per projection (t_e_ = 0.4 s); (**b**) with different exposure time per projection and using a constant number of projections (N_P_ = 1801) per 180° rotation. Data labels refer to scan identifiers; see Table 1.

Figure 5a presents the number of detected contacts resulting from varying the number of projections recorded during XCT scanning, at a constant X-ray exposure time of 0.4 s. The segmentation method used was global thresholding and contacts were extracted by applying a LTF of 1.0 to the presumptive contact areas retrieved from the labelled volume. This graph shows that changing the number of projections resulted in a decrease in the number of spurious contacts detected in the top subvolume with the PE film separator. The sum of spurious contacts decreased from 13 to 8 by increasing projections from 721 to 3601 per 180° rotation, and down to 3 for a full 360° acquisition. However, this number of contacts was well below the original number of apparent contacts retrieved from the label volume, which was consistently above 15 for all four scans in this figure, as presented in Table 2.

Varying the number of projections also altered the number of true contacts detected in the bottom layer without the prescribed separation, also shown in Figure 5a. In this case, the number of detected contacts varied between 45 and 50, with seemingly lower counts with increasing number of projections recorded. A ground truth value of 45 contacts was derived from scan A4 with a large number of projections and a high total X-ray exposure time.

### 3.2. Effect of Exposure Time on Contact Measurements

Figure 5b depicts the effect of varying the exposure time per projection on the number of detected contacts. All scans were acquired using 1801 projections per 180° rotation, the segmentation method used was global thresholding and contacts were extracted by applying an LTF value of 1.0 on apparent contacts detected in the labelled volume. It may be observed that drastically reducing the exposure time had a relatively moderate impact on the number of spurious contacts found for the non-contacting top subvolume, with an increase from 8 at t_e_ = 0.4 to 10 at t_e_ = 0.05 s. This range was again lower than the number of apparent contacts recovered from the labelled volume (Table 2). It should be recalled, however, that the true number of contacts was none, as the pellets were separated by a PE film. A broadly similar trend emerges for the bottom subvolume of contacting particles, with a moderate decrement in the number of detected contacts at the lower exposure intervals.

### 3.3. Effect of Angular Range and Sample Offsetting

**Figure 6:**
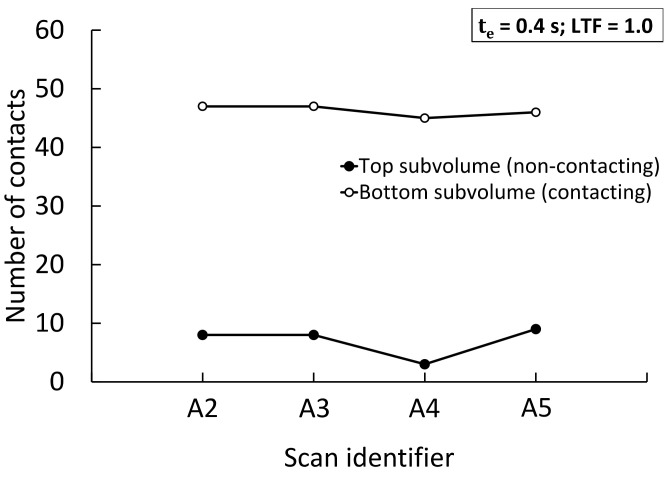
Number of contacts measured on scans acquired using different angular ranges and sample position, as presented in Table 1.

Figure 6 compares the number of detected contacts resulting from acquiring XCT data using a 0–180° angular range (scans A2, A3), a 0–360° angular range (scan A4), and the half-acquisition technique by which the sample is placed at an offset from the COR of the tomography stage and scanned through 360° to increase the field of view (A5; see Section 2.2). As with the data in Figure 5a, scans were acquired using an X-ray exposure time of 0.4 s. Likewise, the conventional global thresholding method without local refinements was employed to binarise the images and contacts were extracted by applying an LTF of 1.0 to presumptive contact regions found in the labelled volume (Table 2). By comparing the results for scan A3 and A4, it may be noted that the doubling of the angular range, and of the number of projections, resulted in a lower number of spurious interparticle contacts detected in the non-contacting subvolume. In contrast, the 0–360° rotation half-acquisition technique delivers a marginally higher contact over-detection number when compared with scan A2 (8 vs. 9 contacts), which was acquired with the same number of projections and, thus, the same total exposure time per 180° rotation. The number of contacts in the bottom subvolume of touching pellets did not vary significantly with the different acquisition methods, generally exhibiting an over-detection of up to two counts, or less about 4%.

### 3.4. Effect of Local Refinement Technique on Contact Measurements

**Figure 7:**
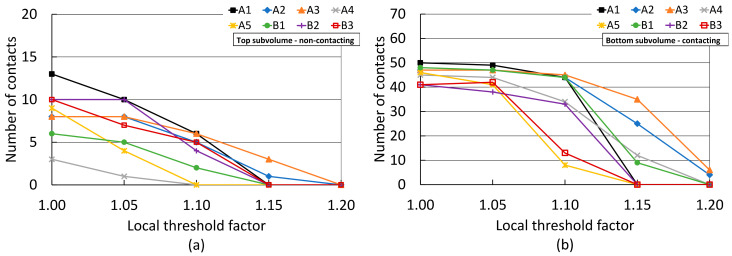
Number of detected contacts between pellet lattices after local refinement using different local threshold factors: (**a**) top subvolume with a prescribed interparticle separation; (**b**) bottom subvolume of full-contacting particles. See Table 1 for scan acquisition details. Data binarised using thresholding.

Figure 7 presents the effect of applying the local refinement technique proposed by Wiebicke et al. [17], summarised in Section 2.4. The XCT data were segmented using global threshold binarisation and LTFs ranging from 1.0 to 1.2 were applied to the apparent contacts extracted from the labelled volume, as described in Section 2.3. In Figure 7a, it may be observed that a local threshold value just 1.05 times larger than the global threshold used to binarise the data was sufficient to produce a notable decrease in the number of spurious contacts found for the top subvolume of film-separated pellets, for most scans. For these scans, this represented a false positive count reduction of between 17 and 67%. However, scans A3 and B2 required larger LTFs values to achieve spurious count decrements. The use of higher local thresholds resulted in similarly substantial reductions in false contacts. Significantly, Figure 7b shows that even the lowest LTF value of 1.05 resulted in the under-detection of true contacts for all scans but A2, A3, and B3. This was particularly severe for the lower exposure scan B2, and for the half-acquisition scan A5. For these scans, an LTF of 1.05 resulted in up to 11% of the true contacts detected with LTF = 1.0 being lost. Moreover, spurious contact elimination in the non-contacting top subvolume could only be achieved for most scans after the use of LTFs of 1.15, which resulted in the drastic under-detection of true contacts in the bottom subvolume, to the point of completely removing all true contacts in half of the XCT datasets. Even the high-exposure scan A3, which was less susceptible to contact suppression by local refinements, exhibited a reduction of true contacts of nearly 26% from the count at LTF = 1.0 when an LFT of 1.15 was used for local refinement.

### 3.5. Effect of Segmentation Method on Contact Measurements

**Figure 8:**
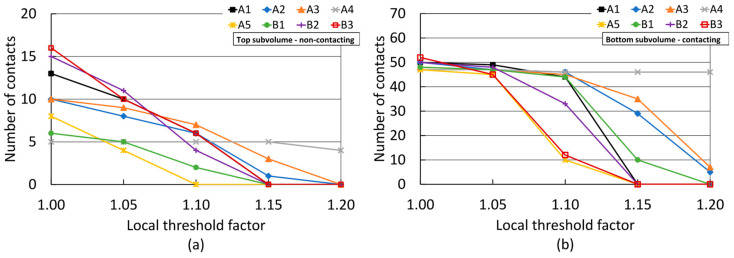
Number of detected contacts between pellet lattices after local refinement using different local threshold factors: (**a**) top subvolume with a prescribed interparticle separation; (**b**) bottom subvolume of full-contacting particles. See Table 1 for scan acquisition details. Data binarised using U-Nets.

Figure 8 presents the outcome of applying the local refinement technique on datasets labelled using U-Net-based binarisation. Figure 8a shows the results for the top subvolume with a prescribed interparticle separation. A comparison with results from thresholding-based segmentation, presented in Figure 7a, suggests that U-Net-based segmentation resulted in a higher spurious contact detection with the application of an LTF value of 1.00 to the apparent contacts of the labelled volume, for most datasets.

The application of local refinements resulted in a sharper decline in false contact counts with the use of an LTF of 1.05 than what was observed for threshold-based results (Figure 7a). The use of higher LTF values resulted in a comparable drop in contact overdetection as that obtained in the threshold-segmented data. As before, most datasets achieved the ground truth zero-contact count at LTF = 1.15, except for datasets A2, A3, and A4, which were less affected by contact refinement. The effect of local refinements on the bottom subvolume of fully contacting particles was also comparable with that observed for the data segmented via thresholding at LTFs values above 1.05. The use of local refinements led to the under-detection of true interparticle contacts, with the error being very large for most datasets with the use of LTF = 1.15 or higher. High-exposure scans A3 and A4 were less susceptible to contact under-detection resulting from local refinements. However, under-detection was notably more moderate in the latter, amounting to just 2% lower at LTF = 1.15 than the number obtained with LTF = 1.0. Furthermore, and in contrast with the threshold-based results, all scans exhibited a light reduction in true contact counts with the use of LTF = 1.05.

## 4. Discussion

### 4.1. Role of XCT Scan Acquisition Parameters and Segmentation Method on Contact Over-Detection

**Figure 9:**
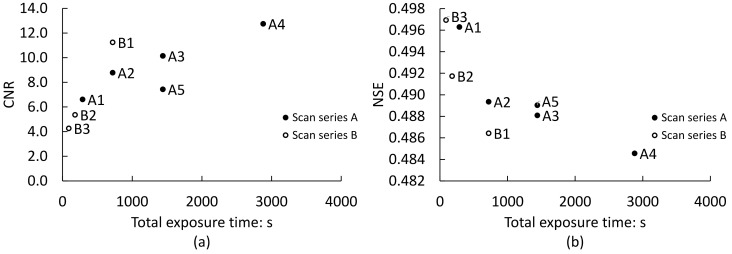
Image quality metrics for the central slice of the bottom (contacting) subvolume: (**a**) contrast-to-noise ratio, CNR (a higher value indicates better image quality); (**b**) normalised Shannon entropy, NSE (a lower value indicates better image quality). See Appendix A for calculation details.

It is remarkable that the labelled subvolume for the film-separated lattices obtained by both segmentation methods returned a large number of false positive contacts, as may be seen from Table 2. This is despite the significant advancement in image quality for scans with higher total exposure times. This is reflected by the improvement in contrast-to-noise ratio (CNR) and normalised Shannon entropy (NSE) image quality metrics with increasing total X-ray exposure times, as presented in Figure 9. These metrics are defined in Appendix A. These results indicate that, without further processing after segmentation and labelling, the use of better quality XCT images may not necessarily pose an advantage with regards to contact detection, and severe over-detection errors may be expected, as originally proposed by Wiebicke et al. [17]. As presented by these authors, such error may be preliminarily attributed to the effect of the partial volume blur rather than image noise.

However, the use of a local threshold at the apparent contacts, even with an LTF = 1.0 (i.e., using the same value as the optimal global threshold), returned a lower spurious contact count, as may be seen by comparing data in Table 2 with results in Figure 7a and Figure 8a. For the data segmented via thresholding, this indicates that the morphological operators used to prepare the data for particle labelling may have contributed to create spurious contacts. Some of these false contacts seem to have been removed during the local assessment procedure, due to local thresholding being applied to the original greyscale data without posterior morphological treatments. For the U-Net segmented data, moderately lower apparent contact counts than those resulting from the global thresholding approach were measured for most datasets without the use of local thresholding (Table 2). However, as these are still higher than the number of counts after local thresholding with LTF = 1.0, it is posed that contact over-detection stemmed from the inability to eliminate spurious contacts in the training data (as no morphological operations were carried out after U-Net binarisation). That is, if spurious contacts could not be effectively removed from the training data by the human operator preparing it, they would persist in the U-Net-segmented data. This explains why highest quality datasets A4 and B1 were the only cases which produced significantly fewer false contacts in the labelled image segmented via U-Nets without local refinements (Table 2). For these datasets, gaps between particles were more easily distinguished by the human operator during manual labelling of training images.

**Figure 10:**
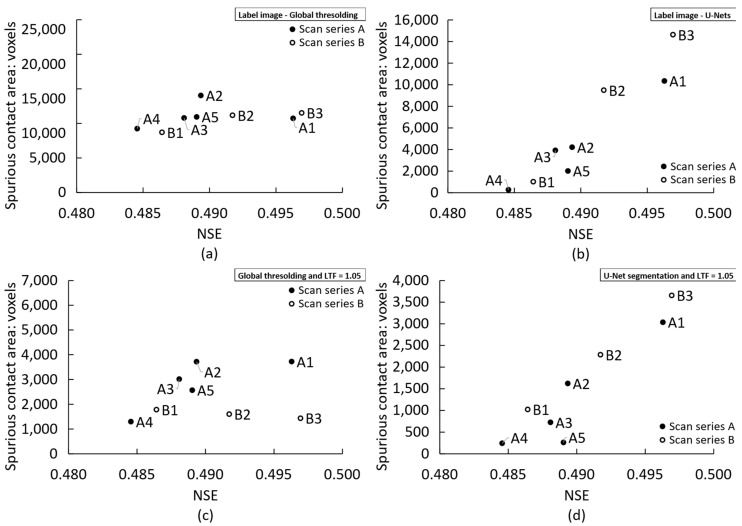
Correlation between spurious contact area and normalised Shannon entropy (NSE) using: (**a**) the global threshold label image; (**b**) the U-Net-derived label image; (**c**) the global threshold label image with local refinements using an LTF of 1.05; (**d**) the U-Net-derived label image with local refinements using an LTF of 1.05.

Furthermore, a comparison between the total spurious interparticle contact area measured for each scan (i.e., before the application of LTF = 1.0 at presumptive contacts, or any other local refinement), presented in Figure 10b,d, reveals that the use of scan parameters that lead to lower acquisition noise (i.e., higher NSE and CNR) in most cases resulted in smaller spurious contact areas for the U-Net-segmented data. This may be associated with the higher segmentation accuracy attributed to CNN-based methods compared with conventional global thresholding approaches, as demonstrated in previous works [23,24]. Nevertheless, it must be again emphasised that such accuracy improvements hinge on the preparation of suitable training label images where partial volume blur is omitted from the grain/solid label. Such images are usually obtained via hand-annotation, which may be an onerous task and limited by the perception of the human operator. Additionally, the implementation of a U-Net method requires significant computing resources.

A similar conclusion regarding the link between image quality and spurious contact area cannot be reached for the threshold segmented data. In this case, contact areas tended to remain relatively constant with image quality, as evidenced in Figure 10a. This trend persisted after the use of the local refinement technique with an LTF of 1.05, as shown in Figure 10c. This occurs because the local refinement technique proportionally cuts down the size of the spurious contact area, and thus, the initial number of voxels forming the false contact in the labelled image controls the number of false contact voxels remaining after local refinement. This also indicates that the amount of image noise plays a minor role in false contact detection when a thresholding technique is used, for both local and global classification purposes. Again, it may be posed that, for this segmentation technique, the paramount factor affecting spurious contact area is the partial volume effect [17].

Conversely, the use of non-thresholding binarisation methods such as U-Nets seems to reduce the importance of partial volume blurring and increases the role of acquisition-dependent image quality. This is evidenced by the reduction in spurious contact areas resulting from the use of U-Net binarisation on higher quality images shown in Figure 10b,d, as discussed previously in this section. It is thus possible that conditions where the partial volume effect is diminished by the lower sphericity and sharper edges of the granular material may result in a preponderant role for acquisition noise on contact detection errors when non-thresholding binarisation methods are used.

Figure 9 also conveys that higher image quality metrics are not solely related to increased total exposure times. Notable increases in image quality emerge from the use of a 0–360° acquisition range for scan B1 (Table 1), when compared with 0–180° range scans with a similar number of projections per half rotation and similar or higher exposure times. For instance, scan B1 exhibits significantly higher image quality metrics than scan A3 with the same number of projections acquired from a half rotation and twice the exposure time per projection. In fact, even with the use of seemingly quality-independent global thresholding, scan A3 returned a similar number of false contacts than scan B1 with half the total exposure time, as shown in Figure 5. This may have two origins. Firstly, the use of full-rotation acquisition may have mitigated truncation artefacts and reduced noise associated with a slight sample offset from the tomography stage COR (see, e.g., [58]). Secondly, the 360 to 180° sinogram conversion step during reconstruction (Section 2.3) encompassed the merging of sinogram overlap regions. For standard 360° scans without COR offsetting, this comprised the entire sinograms of each 180° rotation, which may have contributed to noise reduction. For half-acquisition scans, such as scan A5, the overlap area is often reduced to a few millimetres, and thus this advantage appears to have been lost (Figure 9). Thus, though the half-acquisition technique enabled full-field scanning which suppressed truncation artefacts, this appears to not have compensated for the increase in noise associated with the larger angular intervals for the extended FOV (which results in greater subsampling and further departure from the Nyquist optimum; see Section 2.2).

### 4.2. Critical Assessment of the Local Refinement Technique

**Figure 11:**
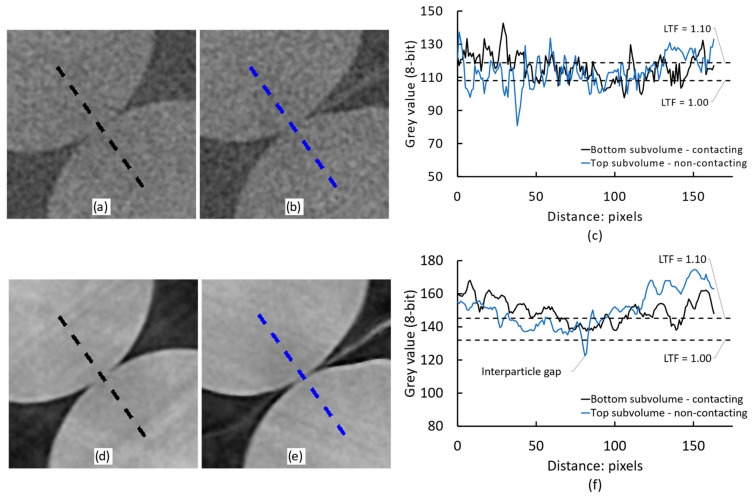
Grey value sampling profiles on horizontal XY slices: (**a**) true interparticle contact in bottom subvolume of scan B3; (**b**) film-separated particles in top subvolume of scan B3; (**c**) grey value profiles sampled from black and blue dotted lines in (**a**) and (**b**), respectively; (**d**) true interparticle contact in bottom subvolume of scan A4; (**e**) film-separated particles in top subvolume of scan A4; (**f**) grey value profiles sampled from black and blue dotted lines in (**d**) and (**e**), respectively.

The local refinement technique effectively suppressed spurious contacts for most datasets with the use of high LTF factors, at the expense of also drastically reducing the number of true contacts, as demonstrated in Figure 7 and Figure 8. This occurred because the application of a higher threshold at interparticle contact regions effectively ‘moves the goalposts’ of what constitutes the solid fraction, under the assumption that false contacts are created by voxels with a higher grey value than the pore space and the global threshold value, but lower than what constitutes the ’true´ solid fraction. Figure 11 plots each threshold level with regards to the grey value profile across a sampling path normal to a true and a film-separated interparticle contact, for scans B3 and A4 with the lowest and highest total exposure times, respectively. For scan B3, it may be seen that the global threshold value used (LTF = 1.0) excluded some noise-induced grey value dips (Figure 11c). This created ‘holes’ or ‘islands’ within the grain label, which were then filled by the morphological processing routines described in Section 2.3. Such drops in grey value were effectively indistinguishable from the actual gap between particles in the lower image quality datasets. As the local threshold increased, e.g., to LTF = 1.10 in Figure 11c, more of the grain fraction was excluded from the grain label, naturally without any preference towards spurious contact voxels. As may be noted, this eliminated both the spurious and true contacts by removing swaths of both particles from the grain label.

Figure 11f conveys that the global threshold value used (LTF = 1.0) solely excluded the gap between the film-separated particles of scan A4 (Figure 11e). It may be seen by comparing with Figure 11c that this was only possible due to the lower amount of noise in the image and the improved contrast, which resulted in such gap exhibiting a grey value that was clearly distinguishable from that of the pellets. Figure 11f shows that the use of a slightly higher local threshold value may lead to a better characterisation of the gap between particles. However, as with the lower quality scan, the application of significantly higher local threshold values, e.g., with LTF = 1.10, may lead to the exclusion of pellet pixels, eventually resulting in the separation of truly contacting particles.

These observations denote that exclusion of solid fraction voxels by the higher local threshold values appears to be root cause of the sharp decline in the number of detected true interparticle contacts resulting from the use of the local refinement technique. Such proclivity to errors persists even when using a relatively modest increment in the threshold value used for local refinements, when applied to low-quality images, and when applied to spheroidal grain packings as in the present study. Considering that reductions in false contacts without sacrificing true counts were achieved by: (1) increasing the quality of the XCT data via optimised acquisition parameters, (2) using a U-Net based binarisation method which benefited from better quality images, and (3) implementing the local refinement technique without increasing the local threshold value, it is proposed that the local refinement technique with LTF > 1.0 may not be an ideal first choice to improve contact detection accuracy.

If the use of the local refinement technique with LTF > 1.0 is deemed necessary, results presented in Figure 7 and Figure 8 suggest that a reasonable reduction in false contacts may be achieved with a limited impact on true contacts if the LTF value remains low and the image quality is high. For the present study, best results were achieved with LTF = 1.05 on high-quality data. Additionally, the risk of suppressing true contacts was lowest when the apparent contacts on which local refinement was applied were extracted from labelled data produced using U-Nets. As explained in Section 4.1, this is attributed to the smaller spurious contact areas resulting from U-Net-based binarisation and the absence of morphological computations prior to watershed-based labelling.

### 4.3. Towards an Optimised Workflow for Contact Detection

From the observations in Section 4.1 and Section 4.2 above, an ideal workflow for contact detection should seek to maximise image quality and segmentation accuracy. Results suggest that the use of the local refinement technique with high local threshold values should be discouraged, considering the high risk of suppressing true contacts and its limited applicability to low-sphericity/high-angularity geomaterials. If the application of local refinements is deemed necessary, the results of this study suggest that the local threshold value to be use should be only slightly higher than the optimum global threshold value for binarisation (i.e., just above LTF = 1.0).

It is recognised that achieving high image quality (i.e., low noise) metrics may be restricted by scan time limitations and throughput requirements, as XCT scan time tends to be expensive. It is thus desirable to achieve the highest image quality possible with the lowest scan time investment. The results of this study suggest that this may be achieved using a full rotation (360°) acquisition technique, as opposed to the half rotation method commonly used in synchrotron XCT.

Considering the above, a tentative workflow for improved contact detection may comprise the following:

1. Full rotation (360°) acquisition using a number of projections as close to the Nyquist optimum as practicable and no less than 35% of this value.
2. The exposure time per projection should deliver suitable transmissivity values, for example, a minimum of 10% as recommended in [59].
3. After reconstruction, mild edge-preserving filtering may be carried out to reduce noise, for example, using an anisotropic diffusion filter.
4. Binarisation should be performed using a high-accuracy method, for example, via CNNs, which may reduce the size of spurious contact areas with increasing image quality. CNNs also seem to be resilient against greyscale variations and artefacts associated with polychromatic XCT scans [50], such as those carried out using laboratory-based X-ray sources. Morphological operations may significantly increase the number and area of false contacts and should be used sparingly or avoided if possible.
5. Particle labelling should then be performed, usually via a watershed-based routine.
6. Apparent contact regions may then be extracted from the labelled image, for example, using the tools provided in the SPAM python library [56,57].
7. Apparent contact regions should thereafter be used to retrieve the corresponding apparent contacts in the XCT greyscale image. If morphological operations were used during segmentation and labelling, local refinement using the optimal global threshold value (i.e., LTF = 1.0) can be used to filter out spurious contacts produced by these procedures. If deemed necessary, a slightly higher local threshold value could be used at the risk of losing a relatively small number of true contacts if image quality is high. There is no evidence that conventional global thresholding with morphological operations, nor U-Net-based binarisation by themselves can deliver acceptable results for spheroidal grain packings.
8. If surplus scanning time is available, it is recommended to acquire additional data using higher imaging specifications (e.g., number of projections, exposure time per projection, rotation range) to obtain higher quality data from which lower quality scans can be assessed.

## 5. Conclusions

The role of acquisition parameters and image processing methods in synchrotron XCT imaging for interparticle contact detection in granular materials was investigated. The objectives of the study were to (1) assess how the number of X-ray projections, the exposure time per projection, the rotation range during acquisition and the position of the sample with regards to the mechanical centre of rotation affect contact detection; (2) assess the effectiveness of a recently developed local thresholding technique for spurious contact mitigation; and (3) assess how a conventional grey value thresholding method compares with a novel U-Net-based binarisation technique with regards to interparticle contact detection accuracy. For this purpose, a hexagonal close-packed arrangement of glass pellets with and without a prescribed separation between lattices was scanned using a targeted range of acquisition parameters, followed by the application of the proposed image processing methods. Significant outcomes of this research were:

1. Varying acquisition parameters leads to different amounts of noise in the reconstructed images, which was measured using the image quality parameters contrast-to-noise ratio (CNR) and normalised Shannon entropy (NSE). The best image quality metrics resulted from the use of a large number of projections with high exposure times, as expected.
2. Image quality metrics may be optimised by recording projections through a full rotation (360°) instead of the customary half-rotation used in synchrotron imaging, with the same total X-ray exposure time. This was associated with the merging of sinograms from each half (180°) rotation during reconstruction.
3. The conventional global thresholding approach involving morphological computations to suppress binarisation noise is unsuitable for accurate interparticle contact detection, regardless of image quality. This method invariably results in the erroneous classification of voxels at the gap between closely spaced particles as part of the solid fraction, leading to spurious contacts. This was attributed to the partial volume effect, as in the literature.
4. The use of U-Net-based segmentation, which did not involve morphological processing, was also incapable of significantly reducing the number of false contacts, except for the highest-quality XCT data. It did, however, deliver smaller spurious contact areas with increasing image quality, suggesting that acquisition-based image noise may play a significant role for this method. The reduction in spurious contact areas was associated to a higher segmentation accuracy, as demonstrated in recent publications. Such improved accuracy resulted in the exclusion from the pellet label of some of the partial volume and noise voxels at the gap between closely spaced particles. However, the preparation of hand-annotated training images where such exclusion was implemented, as well as the use of high-performance computing during training may be operator-dependent and onerous in time and computing resources.
5. The novel local refinement technique, where apparent contacts on the greyscale image are re-assessed by applying a local threshold, was found to suppress a significant number of spurious contacts when the local threshold value was equal to the ideal global threshold. However, while the use of a higher local threshold could eventually eliminate all spurious contacts, it also resulted in the suppression of a very large number of true contacts. This occurred because optimal global threshold values are often selected to include as much of the grain phase as possible while excluding as much of the background, noise, and artefact voxels as practicable. Increasing this threshold value invariably excludes true grain voxels, creating artificial gaps between particles.
6. It was proposed that an optimal workflow for interparticle contact detection should include the use of the novel local refinement technique without the use of high local threshold values. The workflow should also seek to maximise image quality (i.e., lowering noise) and include the application of non-thresholding segmentation approaches, such as those based on U-Nets or other convolutional neural networks, without the use of morphological operations. A compromise between lowering scanning times and achieving high image quality metrics may be reached by using techniques such as full-rotation acquisition with sinogram merging during reconstruction.

## Figures and Tables

**Table 1:** XCT scan parameters and threshold value for binarisation (Section 2.3).

Scan	Angular Range (°)	Number of Projections	Exposure Time Per Projection (s)	Total Detector Exposure Time (s)
A1	0–180	721	0.4	288.4
A2	0–180	1801	0.4	720.4
A3	0–180	3601	0.4	1440.4
A4	0–360	7201	0.4	2880.4
A5 ^1^	0–360	3601	0.4	1440.4
B1	0–360	3601	0.2	720.2
B2	0–180	1801	0.1	180.1
B3	0–180	1801	0.05	90.0

^1^ Half-acquisition scan.

**Table 2:** Number of apparent contacts detected on top (non-contacting) labelled subvolume before local refinements, for each segmentation method.

Scan	Global Thresholding	U-Nets
A1	17	17
A2	17	15
A3	16	15
A4	15	5
A5 ^1^	15	11
B1	15	6
B2	17	17
B3	19	18

^1^ Half-acquisition scan.

**Figure A1:**
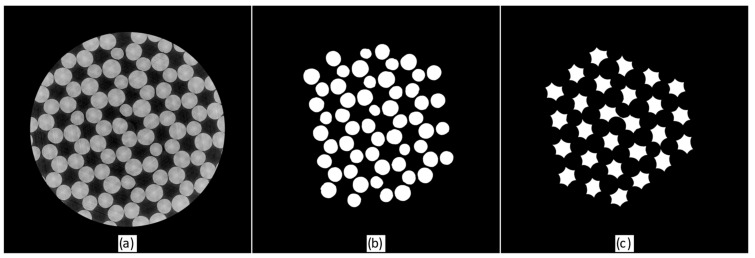
Extraction of ROI areas used to compute CNR metrics: (**a**) original XCT central slice of the bottom subvolume (contacting) of scan A4; (**b**) pellet foreground; (**c**) pore space background.

## Data Availability

The XCT data presented in this study are openly available in Zenodo at https://doi.org/10.5281/zenodo.5806270, reference number [47].

## Appendix A

Contrast-to-noise ratio (CNR) and normalised Shannon entropy (NSE) are conventional image quality metrics [60,61]. The CNR is defined as:

(1) $$\mathrm{CNR}=\frac{{\bar{\mathrm{GV}}}_{f}-{\bar{\mathrm{GV}}}_{b}}{\sigma_{b}},$$

where and are the mean grey values of the foreground and background, respectively, and σ_b_ is the standard deviation of the background. A higher CNR theoretically denotes better image quality. Similarly, the NSE is computed as:

(2) $$\mathrm{NSE}=\left(-\frac{1}{\log_{2}N}\right)\sum_{i}^{N}p_{i}\log_{2}\mathrm{pi},$$

where i = 1… N refers to histogram bins with pixel/voxel counts and p_i_ is the number of histogram counts per bin. A lower value nominally denotes better image quality.

Both the CNR and NSE were computed for the middle slice of the bottom subvolume of contacting particles, over the central 3280-pixel diameter ROI used for contact analysis. For the CNR, the foreground and background regions were the eroded areas corresponding to pellets and pore space, respectively, as shown in Figure A1.
